# Quantifying the Changes of Mechanical and Electrical Properties of Paralyzed Muscle in Survivors With Cervical Spinal Cord Injury

**DOI:** 10.3389/fneur.2021.720901

**Published:** 2021-09-08

**Authors:** Huijing Hu, Yingyue Chen, Xiaoyun Wang, Wai Leung Ambrose Lo, Le Li

**Affiliations:** ^1^Institute of Medical Research, Northwestern Polytechnical University, Xi'an, China; ^2^Guangdong Work Injury Rehabilitation Center, Guangzhou, China; ^3^Department of Rehabilitation Medicine, The First Affiliated Hospital, Sun Yat-sen University, Guangzhou, China

**Keywords:** spinal cord injury, muscle tone, electrical impedance myography, muscle strength, clinical scales

## Abstract

**Background:** Survivors with spinal cord injury (SCI) have neuromuscular deficits such as muscle atrophy that lead to functional impairments. This study utilized myotonometry and electrical impedance myography (EIM) to quantitatively evaluate the changes in muscle mechanical properties and compositions after SCI.

**Methods:** This study adopted a cross-sectional design. Eighteen SCI patients and 18 healthy individuals were recruited. The outcome measures were: (1) The myotonometer measured muscle mechanical parameters of oscillation frequency (freq), dynamic stiffness, logarithmic decrement (decr), mechanical stress relaxation time, and indication of creep. (2) The electrical impedance myography measured parameters of resistance (R), reactance (X), and phase angle (θ). (3) muscle strength (maxForce); (4) clinical scales of Manual Muscle Testing (MMT) and modified Ashworth scale (MAS). All outcome measures were compared between the bicep brachii muscle of the weaker side of the SCI group and the non-dominate side of the healthy group. Correlation analysis was performed at quantitative data and clinical scales.

**Results:** Freq, stiffness, and maxForce of the SCI group were significantly lower (*p* < 0.01) than those of the healthy control. The relaxation time and creep were significantly higher in the SCI group than in the control group. Significant differences of R and Xc were observed between the two groups. Significant correlation was observed between freq, stiffness, and months past injury, and between Xc, creep, and relaxation time.

**Conclusions:** Reduced muscle tone and stiffness might relate to muscle atrophy, and higher relax time and creep may be caused by poor contractile ability. The changes in EIM parameters could indirectly reflect the muscle cell size, and fatty and connective tissue alterations. These findings support the feasibility of myotonometer and EIM to quantify muscle mechanical and intrinsic properties in patients with SCI. The results could facilitate the understanding of neuromuscular changes that are related to functional impairments.

## Introduction

Spinal cord injury (SCI) refers to the damage of the structure or function of the spinal cord that results in a different extent of motor, sensory, and autonomic dysfunction below the injured level (1). SCI-related functional impairment brings heavy burden to patients, families, and society. Cervical spinal cord injury (CSCI) is a common type of SCI, accounting for ~55% to 75% of the total SCI prevalence (2). Muscle atrophy, spasticity, and learned disuse are common among patients with SCI, which contribute to motor function impairment (3, 4).

Muscle atrophy is related to the damage of the motor neuron, which contributes to the inactivity of the affected skeletal muscles, leading to changes in the muscle mechanical load conditions and muscle length shortening due to sarcomere loss (5). Muscle atrophy also resulted in a change in the proportion of the type of muscle fibers, with the amount of type I fiber typically increased, whereas the amount of type II fiber reduced (6). Muscle atrophy also contributes to a decrease in cross-sectional area of the muscle below the injury level (7) and an increase in intramuscular adipose tissue (3). The increase in intramuscular adipose tissue and changes in muscle composition are related to muscle weakness (8). The quantitate evaluation of atrophy-related muscle dysfunction tends to rely on the clinical presentation as assessed by the assessor. This include manual palpation, manual muscle test, or the clinical scales such as modified Ashworth scale. These assessment methods are subjective and lack reliability or validity. Thus, quantitative evaluation methods to assess the change in muscle properties in patients with SCI are in great need to monitor disease progression and evaluate the impact of intervention.

Common complications of patients with SCI such as muscle wastage, muscle contracture, and muscle atrophy contribute to the alteration of muscle mechanical properties. The muscle mechanical properties of tone and stiffness are considered to be fundamental in energy-efficient muscle contraction (9). Upper limb muscles of the bicep brachii and brachioradialis muscles are particularly important due to their frequent involvement in activities of daily living such as feeding, door opening, and dressing (10). Interventions often aim to improve functional outcomes by reducing muscle spasticity (11). Despite the importance of the muscle tone and stiffness, these two aspects are often clinically assessed by functional scale such as the modified Ashworth scale or by manual palpation (12). In recent years, there is an increasing number of publications that supported the measurements of handheld myotonometer, which are valid and reliable to quantify the muscle mechanical properties. Handheld myotonometer measures the mechanical vibration of soft tissues caused by the mechanical impulse induced by the device (13). The probe of the device applies multiple short mechanical impulses to the skin surface of the tested muscle to generate damped oscillations (14). The device then calculates the parameters of muscle mechanical properties, which are oscillation frequency (freq), dynamic stiffness, logarithmic decrement (decr), mechanical stress relaxation time (relax), and indication of creep (creep). Oscillation frequency characterizes the muscle tone or intrinsic tension of a muscle. Muscle stiffness characterizes the resistance to a contraction or an external force that deforms its initial shape. Decr refers to the logarithmic decrement of the natural oscillation of a muscle. It indicates the elasticity and dissipation of the mechanical energy of the muscle when tissue recovers its shape from being deformed. Relaxation time is the time for the muscle to restore its shape from deformation after a voluntary contraction or an external force is removed. Creep is the gradual elongation of a muscle over time when placed under a constant tensile stress. It is the ratio of the relaxation and deformation time of the muscle. The myotonometer has been applied to assess muscle mechanical properties in patients with neurological and musculoskeletal diseases, including Parkinson's disease (15), stroke (16–18), low back pain (19, 20), and Achilles tendinopathy (21). In addition to alteration of muscle mechanical properties, the intrinsic properties of muscle composition are also affected in patients with SCI.

Muscle composition could be monitored non-invasively by electrical impedance myography (EIM). EIM applies low-intensity, high-frequency current to the localized muscle area and measures the voltage and current that pass through the tissues. Muscle health could then be evaluated from the electrophysiological conditions of muscle tissue by calculating tissue compliance and resistance (22). The three most commonly used EIM parameters are resistance (R), reactance (Xc), and phase angle (θ), calculated as θ = arctan (X/R) (23). Electrical impedance myography has the potential to be a biomarker of SCI, given that pathological changes (such as muscle atrophy, muscle fiber denervation/reinnervation, and fatty infiltration) will collectively influence normal impedance characteristics (24). Two published studies indicated quantifiable differences in the muscle composition of the biceps and hand muscles between the stronger and weaker side in patients with SCI, and between patients with SCI and healthy individuals (25, 26).

To date, the majority of the literature assessed the feasibility of the application of EIM to detect muscle composition in patients with SCI. It remains unclear if muscle composition may be related to muscle mechanical properties, and whether changes in muscle composition and muscle mechanical properties are related to clinical measurements in patients with CSCI. This study aimed to apply EIM technology and myotonometer to assess muscle function, and explored their potential relationships with clinical measurements. Findings of this study would provide further understanding of the alteration of muscle health and may form the basis of targeted rehabilitation strategies to improve functional outcome.

## Methods

### Study Design and Sample Population

This study adopted a cross-sectional design to compare the difference in muscle composition, muscle mechanical properties, and muscle force between patients with SCI and healthy individuals. The study also investigated the association between muscle composition, muscle mechanical properties, and muscle strength in patients with SCI. The inclusion criteria were as follows: (1) meet the diagnostic criteria of the American Spinal Cord Injury Society in 2000; (2) traumatic CSCI confirmed by magnetic resonance imaging; (3) medically stabled CSCI patients who underwent cervical spine internal fixation procedure; (4) intact motor function prior to SCI injury, without bone tuberculosis and bone malignant lesions; and (5) able to follow instructions and provide informed consent. The exclusion criteria were as follows: (1) minors; (2) fractures of other body parts other than SCI; (3) existing neurological, musculoskeletal, respiratory, and psychological disorders; and (3) patients who were unable to cooperate. Healthy participants who had no history of neuromuscular disease or other neurological disorders were recruited for the control group. The experimental procedures were conducted in accordance with the Declaration of Helsinki. The study protocol was approved by the Ethical Committee of the Guangdong Industrial Injury Rehabilitation Hospital, Guangzhou.

### Outcome Measurements

#### Electrical Impedance Myography

**Figure 1:**
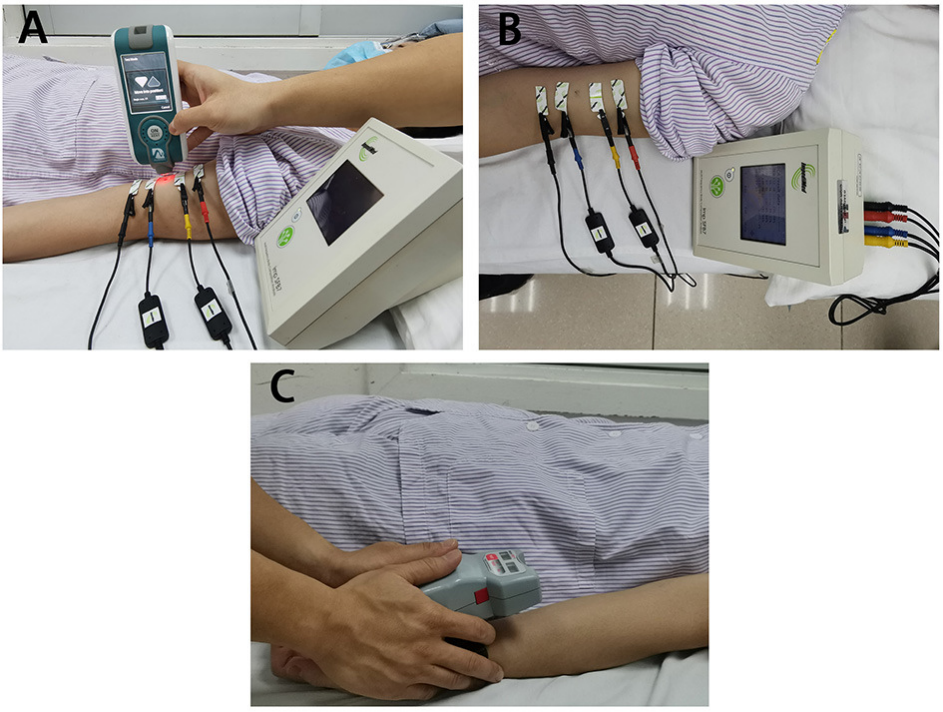
The apparatus for measurement adopted in the study. **(A)** The test location and electrical impedance myography, **(B)** Myotonometer to measure muscle mechanical properties. **(C)** Handheld dynamometer to record muscle force.

The electrical resistance properties that reflect the muscle composition of the bicep brachii were assessed by EIM (Imp SFB7 Impedimed, Inc., Sydney, NSW, Australia). The center of the electrodes was identified as the middle distance between the acromion process and the medial border of the cubital fossa. B-mode ultrasound scan was conducted to confirm the location of the muscle belly and the direction of the muscle fibers of the bicep brachii. Two pairs of electrodes were linearly arranged along the muscle fiber direction, including one pair of voltage electrodes on the inner regions and an outer pair of current electrodes (27). Each pair of electrodes was distributed symmetrically along the center point marked in advance. The dimension of the electrodes was 13 × 10 mm. The distances between the two outer current electrodes and the two inner voltage electrodes were 60 and 20 mm, respectively. Three measurements were recorded at each assessment, and the mean value of the measurement was included for statistical analysis. The data recorded by the device was exported for offline analysis by the Bioimp software (28). The parameters of resistance (R), reactance (Xc), and phase angle (θ) were recorded across multiple frequencies of between 5 and 1,000 kHz, and the parameters recorded at 50 kHz were included in the analysis (Figure 1A).

#### Myotonometer

A handheld myotonometer (MyotonPRO®, Tallinn, Estonia) was applied to quantify the mechanical muscle properties of the bicep brachii. The patients laid in supine with upper extremities relaxed by the side. The wrist joint was kept in the neutral position, and the elbow joint was in full extension. Since muscle tissue is affected by gravity, it is recommended to place the probe perpendicularly to the skin surface (29). The probe of the device was placed on the skin surface of the thickest part of the bicep brachii (Figure 1B). The device was set in triple scan mode where three mechanical impulses (1 s apart) were delivered. A controlled pre-load for initial compression of the subcutaneous tissue was applied before imposing impulses of mechanical force. Once the required depth was reached (indicated by a change in indicator light from red to green), the device then applied three consecutive impulses to induce damped oscillations within the muscle bulk. The average, standard deviation, and coefficient of variation of the three measurements (one test set) were then displayed on the screen. All measurement sets with a coefficient of variation of more than 3% was re-measured. The oscillation pattern recorded by the transducer was used to calculate the muscle mechanical properties (18, 20), which are the parameters of freq, stiffness, relaxation time, creep, and decr (30).

#### Handheld Dynamometer

Muscle force was recorded by the handheld dynamometer (Microfet3, Hoggan Scientific LLc, Salt Lake City, UT, USA) placed on the wrist joint. Subjects were then asked to perform maximum isometric contraction of elbow flexion and maintained the position when maximum force was applied (see Figure 1C). During the test procedure, subjects were asked not to lift any body parts away from the bed. The maximum force (maxForce) was included in the statistical analysis. maxForce was calculated as the average value of three measurements from handheld dynamometer where participants were asked to perform maximum contraction of the elbow flexor muscle. Rest periods were provided during the test procedure to avoid fatigue.

#### Clinical Evaluations

A professional physiotherapist conducted the clinical test of ASIA impairment assessment, Manual Muscle Testing (MMT), and modified Ashworth Scale (MAS). The therapist was blinded to the results of the EIM, myotonometer, and dynamometer. Anthropometric characteristics of the sample population were collected prior to the start of the study. According to the MMT results of the biceps muscles of the patient, the upper limbs were classified as the stronger side (the side with higher MMT score) and weaker side. If the MMT scores on both sides are equal, the dominant side was considered as the stronger side, and the non-dominant side was considered as the weaker side (determined by the self-report of the subject and clinical evaluation). Participants were asked to lie on the treatment bed in a relaxed supine position, with both upper limbs parallel to the body. Then the biceps were evaluated by myotonometer, EIM, and handheld dynamometer. Only the biceps muscle of the weaker side of SCI patients and the non-dominant side of healthy control were recorded.

### Data Analysis

Data analysis was conducted in SPSS 22 software (IBM, New York, NY, USA). Descriptive statistics were calculated for all dependent variables. All parameters were tested for normality by the Chi-square goodness of fit test. Parameters that were normally distributed are reported as mean and standard deviation, whereas median and interquartile range (IQR, between 25 and 75% quartile) were reported for non-normally distributed data sets. All parameters of the weaker side of the SCI patients were paired with those of the non-dominant side of healthy controls to assess the difference in muscle characteristics. Paired *t-*test was conducted for the parameters that follow a normal distribution, and Wilcoxon rank sum test was conducted for parameters that deviate from the normal hypothesis. The correlation between muscle parameters and clinical functions were assessed by either Pearson or Kendall's correlation analysis, depending on normality. For all statistical tests, a two-sided test was performed with statistical level set at *P p* < 0.05.

## Results

Eighteen patients with SCI (2 females and 16 males, mean age 38.94 ± 13.1 years old, all right handed) were recruited in this study. The neurological injury level of the patients ranged between C4 and C6, and the American Spinal Injury Association (ASIA) impairment levels ranged between A and D. The clinical characteristics of the SCI group are presented in Table 1. Eighteen healthy individuals (1 female and 17 males, age 38.04 ± 9.62 years old, one left handed) were recruited in the control group. All of the participants provided written informed consent (or consent form signed by family members for subjects who were unable to write) prior to study enrollment. Subject 18 of SCI did not complete the dynamometer test at the first measurement due to muscle weakness when he was in the early stage of injury. Results present here are based on the data collected from all the participants.

**Table 1:** Baseline characteristics of the patients with spinal cord injury (SCI).

**ID**	**Age**	**Gender**	**Months past injury**	**ASIA impairment scale**	**Neurological level**	**Motor level**		**Sensory level**		**Motor score**		**MMT (BB)**		**MAS (BB)**	
						**L**	**R**	**L**	**R**	**L**	**R**	**L**	**R**	**L**	**R**
1	31	Female	14.5	C	C5	C5	C5	C5	C5	13	8	5	5	0	0
2	62	Male	24	C	C4	C4	C4	C4	C4	26	12	4	4	1+	1
3	23	Male	11.3	A	C4	C4	C4	C4	C4	5	5	5	4	0	0
4	53	Male	5.2	C	C4	C5	C5	C4	C4	27	24	4	5	1	1
5	53	Male	5.5	C	C4	C4	C4	C4	C4	12	14	3	3	2	2
6	30	Male	3.6	A	C6	C5	C5	C7	C6	4	4	4	4	1	1
7	43	Male	11.5	C	C4	C6	C5	C4	C4	34	13	5	4	0	0
8	47	Male	14.1	D	C4	C4	C4	C4	C4	38	38	4	4	0	0
9	22	Male	6.1	A	C5	C5	C5	C5	C5	5	5	5	5	0	0
10	27	Female	15	C	C5	C5	C5	C5	C5	8	8	4	4	0	0
11	43	Male	5.3	B	C4	C4	C5	C4	C5	3	5	3	3	0	0
12	45	Male	11.5	C	C4	C4	C4	C4	C4	13	14	3	4	0	0
13	57	Male	7.7	D	C5	C5	C5	C5	C5	38	38	5	5	0	0
14	49	Male	4.9	A	C6	C6	C6	C6	C6	8	8	4	4	1	1
15	22	Male	10.7	B	C5	C5	C5	C5	C5	11	11	5	5	0	0
16	24	Male	13.7	B	C6	C7	C6	C8	C8	10	9	5	5	0	0
17	30	Male	27.8	C	C4	C5	C5	C4	C4	33	31	4	4	0	0
18	40	Male	2	A	C4	C4	C4	C4	C4	1	1	2	1	0	0

*BB, biceps brachii; MMT, manual muscle test; MAS, modified Ashworth scale*.

### Differences Between Spinal Cord Injury and Healthy Control

**Figure 2:**
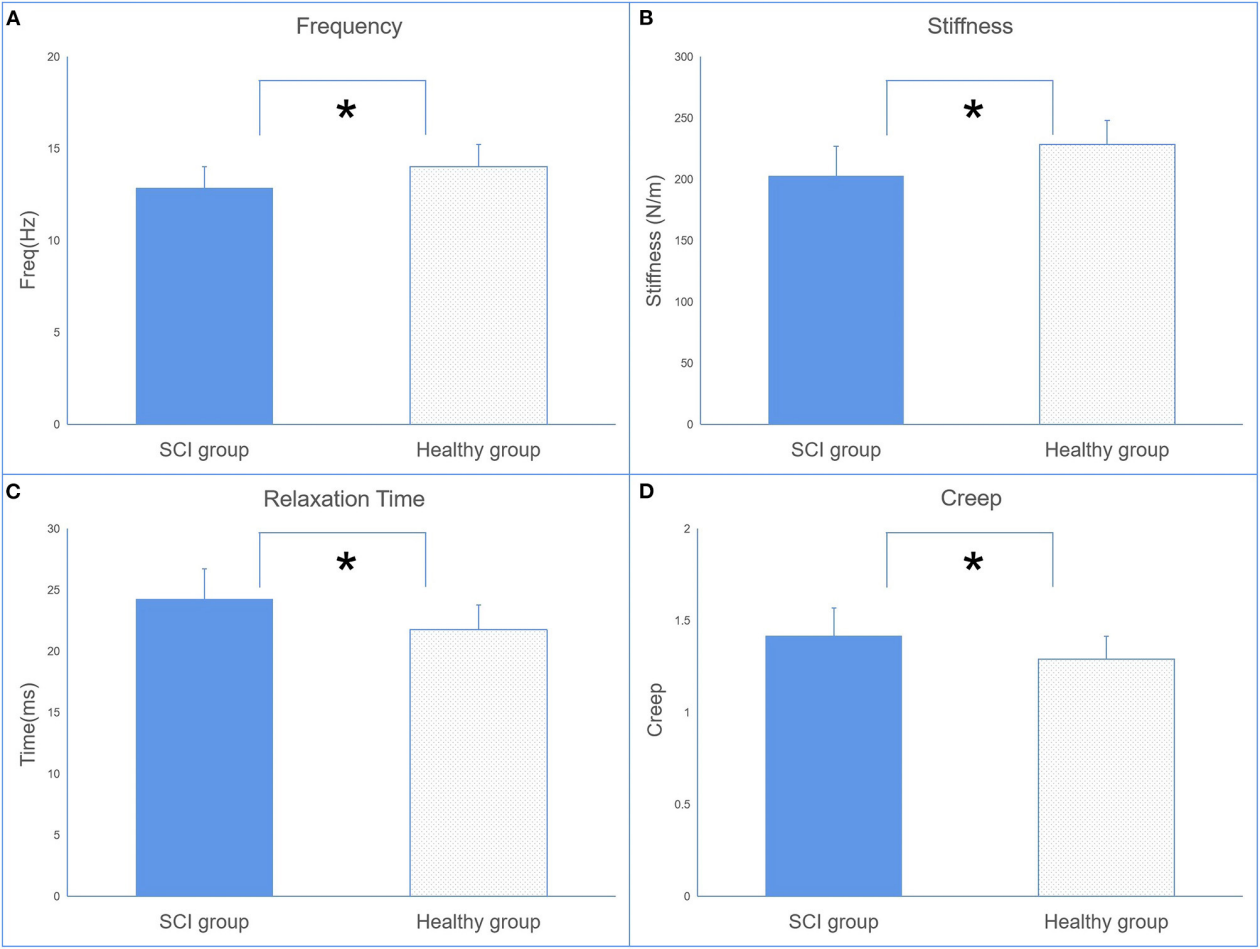
The comparisons of muscle mechanical properties [oscillation frequency **(A)**, dynamic stiffness **(B)**, mechanical stress relaxation time **(C)**, and indication of creep **(D)**] between the SCI and control groups (mean ± SD, * indicates *p* < 0.05 between groups).

Figure 2 illustrates the comparison of myotonometer and dynamometer between the SCI and control groups. The results indicated significant differences in freq, stiffness, relaxation time, creep, and maxForce between the two groups. The myotonometer parameters of freq and stiffness, and dynamometer parameter of maxForce were significantly lower in the SCI group (freq = 12.86 ± 1.14 Hz; stiffness = 202.89 ± 23.48 N/m, maxForce = 24.46 ± 13.70 lb) than in the control group (freq = 14.02 ± 0.86 Hz; stiffness = 228.44 ± 23.81 N/m, maxForce = 41.19 ± 12.30 lb). The relaxation time and creep of myotonometer parameters were significantly higher in the SCI group (relax = 24.27 ± 2.38 ms; creep = 1.42 ± 0.15) than in the control group (relax = 0.21.76 ± 1.76 ms; creep = 1.29 ± 0.11).

**Figure 3:**
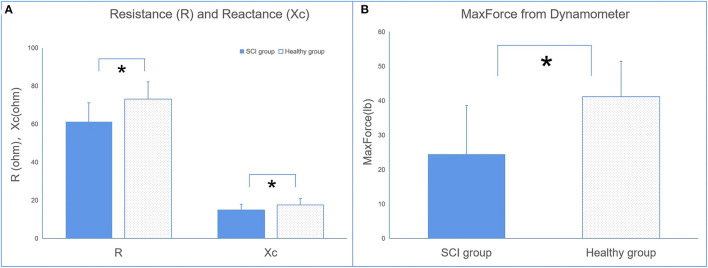
The comparisons of electrical impedance myography (EIM) **(A)** and dynamometer **(B)** parameters between the SCI and control groups (*p < 0.05 between groups).

The EIM parameters of R and Xc were significantly lower in the SCI group (*R* = 61.32 ± 9.63, Xc = 14.99 ± 2.92) than in the control group (*R* = 73.06 ± 8.62, Xc = 17.55 ± 3.07) recorded at 50 kHz (Figure 3A). No significant difference in θ was observed between the two groups.

The results of the dynamometer indicated that maxForce was significantly lower in the SCI group than in the healthy group.

### Correlation Between Parameters

The results of the correlation analysis are presented in Table 2. The parameters of freq, stiffness, relaxation time, and creep of the biceps brachii in the SCI group were significantly correlated with months past injury. Relaxation time and creep are significantly correlated with maxForce, and also with Xc. Θ and Xc are significantly correlated with MMT. Xc is also significantly correlated with maxForce.

**Table 2:** The results of correlation analysis between myotonometer, electrical impedance myography (EIM), and dynamometer.

	**Months post injury**		**Force**		**MMT**		**MAS**		**θ**		**R**		**Xc**	
**Parameters**	***r***	***p***	***r***	***p***	***r***	***p***	***r***	***p***	***r***	***p***	***r***	***p***	***r***	***p***
Freq	0.56	0.02^*^	0.42	0.81	0.18	0.36	−0.07	0.77	0.39	0.37	0.08	0.76	0.50	0.03^*^
Stiffness	0.52	0.03^*^	0.47	0.47	−0.14	0.48	0.05	0.85	0.27	0.27	−0.41	0.87	0.27	0.28
Decr	0.17	0.49	0.15	0.55	−0.32	0.11	−0.01	1.00	−0.37	0.13	0.06	0.84	−0.38	0.12
Relax	−0.72	0.00^*^	−0.59	0.01^*^	−0.38	0.06	0.21	0.31	−0.40	0.10	−0.32	0.20	−647.00	0.00^*^
Creep	−0.67	0.00^*^	−0.51	0.03^*^	−0.49	0.01^*^	0.27	0.19	−0.38	0.12	−0.39	0.11	−0.68	0.00^*^
θ	0.19	0.45	0.45	0.06	0.41	0.03^*^	0.16	0.44						
R	0.38	0.12	0.17	0.49	0.13	0.54	−0.43	0.03^*^						
Xc	0.46	0.06	0.62	0.01^*^	0.59	0.00^*^	−0.14	0.50						

* *p < 0.05*.

## Discussion

This study assessed the mechanical properties, muscle composition, and functional changes in CSCI-related muscle weakness by applying myotonometer, EIM, dynamometer, and clinical scales. The results indicated that mechanical properties and muscle composition were significantly altered when compared with healthy control, and some quantitative muscle measurements correlated with clinical evaluation.

### Quantitative Changes in Muscle Mechanical and Electrophysiological Parameters in Spinal Cord Injury

#### Muscle Mechanical Property Measurement

Methods to quantify mechanical muscle properties are essential to assess the magnitude of muscle abnormality and enable evaluation of effectiveness of intervention (31). This study observed significant differences in freq and stiffness between the weaker side of SCI and the non-dominant side of healthy control as assessed by a myotonometer (Figures 2A,B). As this is among the first study that applied myotonometer to assess muscle mechanical properties in patients with SCI, minimal data are available for direct comparison on the measured parameters. Published literature indicated that SCI patients often suffered from muscle atrophy due to damage of the central nervous system. Several published literature indicated that substantial muscle atrophy took place within 6–12 weeks postinjury (3) and continues afterward if adequate intervention was not provided (32). Findings from the myotonometer measurement indicated that both muscle tone and muscle stiffness are significantly reduced when compared with the healthy participants, suggesting the presence of muscle atrophy. This provides some support on the feasibility of myotonometer to quantify muscle mechanical properties in patients with SCI.

The parameters of relaxation time and creep in the SCI group are also significantly higher than in healthy individuals. This finding suggests a contractile ability in the muscles of SCI. Relaxation time refers to the time it takes for a muscle to return to its shape prior to deformation or a contraction, whereas creep refers to the gradual elongation of muscle tissue over time when placed under stress (14). These properties are related to the contractile ability of the muscle, since skeletal muscle blood supply is dependent on its ability to return to its original shape between contractions (14). This theory is given some support by the result from the dynamometer that patients with SCI have less maximum force than the healthy group (Figure 3B).

#### Electrical Impedance Myography

To date, two published studies were found during our literature search that provided direct evidence to support the change in muscle composition of the biceps brachii and hand muscles in patients with spinal cord injury (25, 26). These findings supported that Xc and R could be the biomarkers in the assessment of muscle intrinsic properties. The parameters of R and Xc are not just related to the muscle mass and muscle fiber geometry but also related to tissue components such as the extracellular and intracellular content of water, as well as the cell membranes properties (33). The alteration in impedance parameters are linked with the alteration in the muscle fiber and damaged cell membrane. These alterations are a result of a reduction in number of fibers and fiber cross-sectional area of muscle tissues, as well as an increase in intramuscular extracellular matrix (23, 24).

There were significant differences in the EIM parameters of X between the SCI and healthy groups. This is consistent with published data (25). No significant difference in R between the SCI and healthy groups was previously reported, which contradicts the finding of the present study. A potential explanation for the contradictory results of R observed between the findings of the resent study and the findings of Li et al. (25) may be due to the difference in the duration of injury of the SCI population between studies. The SCI population in the present study had a shorter duration of injury compared with the other two studies. Therefore, the sample populations may have different degrees of muscle atrophy, which may affect the parameter of R. A published study reported an increased level of intramuscular lipid content in the lower limbs of SCI patients (34). The increase in intramuscular lipid reduces the water content within the muscle tissue itself as water content is higher in muscle tissues. However, during the process of muscle atrophy, muscle tissues may be replaced by fatty tissue and connective tissue, which then increases R. Thus, the *R*-value may be affected by the extent of muscle atrophy or duration of injury.

### Clinical Correlation and Significance of Quantitative Muscle Parameters

The correlation analysis supports the poor muscle contractile ability in patients with SCI and provides further evidence on the feasibility to apply myotonometer to assess muscle function. Both the relaxation time and creep were negatively associated with MMT, with creep reaching statistical significant level, and relaxation time was marginally close to statistical significance. This also corroborates with the significant correlation between relaxation time and maxForce, and between creep and maxForce. All of the myotonometer parameters, except for decr, were significantly correlated with months poststroke. This provides some insight that the mechanical properties may continue to deteriorate for an extended period of time postinjury, possibly due to muscle atrophy. Previous studies reported that the freq measurement of myotonometer was related to the MAS score in stroke survivors (35). This study did not observe a significant correlation between the MAS score and myotonometer. This is due to the small range of MAS score of the SCI group (between 0 and 1), which is likely to result in a low correlation coefficient.

One of the novelties of the present study is the comparison between mechanical properties and muscle composition. The EIM parameters of Xc were found to be correlated with relaxation time and creep. X was previously reported to reflect the damage to a cell member and is particularly prone to be affected by myocyte atrophy (36). A study conducted in mice models that utilized magnetic resonance imaging reported that Xc could be distinguished between healthy and diseased mice (37). As creep and relaxation time are the mechanical properties that reflect muscle contractile ability, they are also likely to be associated with Xc, which represents the state of myocyte atrophy. These findings provide further evidence to support that muscle mechanical properties are complimented by muscle composition.

The correlation analysis indicated that Xc and θ are both significantly related to MMT. Xc was significantly related to maxForce, and θ was marginally significantly related to maxForce. θ is the time shift of sinusoidal wave when traveling through a muscle structure (33). Thus, a reduction in cell size and the presence of increased amount of connective tissue or fatty tissue contribute to a smaller phase angle (22). Thus, the amount of force generated is positively associated with θ.

### Limitations

The results of the present study should be interpreted with caution due to its limitations. First, this preliminary study was not power calculated and may contain type II error. Type II error occurs when accepting the null hypothesis when it is false (38). The sample population contains an uneven number of male and female participants, which might be a confounding factor as a previous study indicated a difference in R, Xc, and θ between men and women (39). However, the control subjects were age and gender matched, which might minimize the effect of this confounding factor. The present study did not adopt the standardized motor examination position of SCI when assessing muscle strength. This was to ensure consistency with the body position adopted for the recording of muscle composition. Body position was reported to affect the measurement of muscle mechanical properties (40). Thus, keeping the body position consistent would minimize the impact of confounding factor. As a preliminary study, our aim was to apply EIM technology and myotonometer to assess muscle function and relationship with clinical measurements in patients with SCI. The effects of different injury levels or ASIA level on muscle composition and muscle mechanical properties were not considered at the current stage. Future studies should recruit a sufficiently large sample population to enable subgroup analysis based on different injury levels. This would enable the investigation on how muscle composition and muscle mechanical properties may vary in patients with different levels of injury and assess if the relationship between EIM myotonometer parameters may be influenced by different levels of injury.

## Conclusion

The findings of the present study demonstrated the feasibility to combine myotonometer and EIM technique to assess the muscle mechanical properties and muscle composition alteration in patients with SCI. Reduced muscle tone and stiffness might be related to muscle atrophy, and higher relax time and creep may be caused by poor contractile ability. EIM changes could indirectly reflect the muscle cell size, and fatty and connective tissue alterations. Some of the muscle mechanical properties are associated with muscle composition, which are also correlated with muscle strength. The findings of this study could facilitate further understanding in neuromuscular changes to functional impairments.

## Data Availability Statement

The datasets analyzed during the current study are available from the corresponding authors upon reasonable request.

## Ethics Statement

The studies involving human participants were reviewed and approved by the Ethical Committee of the Guangdong Work Injury Rehabilitation Center, Guangzhou. The patients/participants provided their written informed consent to participate in this study.

## Author Contributions

HH, LL, and WL conceived and designed the study. HH, YC, and XW performed the experiments. HH, YC, and WL drafted the paper. WL, XW, and LL made contributions to the experiments. WL and LL reviewed and edited the manuscript. All authors had read and approved the manuscript.

## Funding

This study was supported by the Natural Science Foundation of China (Nos. 81702227, 32071316, and 81971224) and partly supported by the Guangdong Basic and Applied Basic Research Foundation (No. 2020A1515011356), Guangzhou Research Collaborative Innovation Projects (No. 201907010034), and the Non-profit Central Research Institute Fund of Chinese Academy of Medical Sciences (No. 2020-JKCS-005).

## Conflict of Interest

The authors declare that the research was conducted in the absence of any commercial or financial relationships that could be construed as a potential conflict of interest.

## Publisher's Note

All claims expressed in this article are solely those of the authors and do not necessarily represent those of their affiliated organizations, or those of the publisher, the editors and the reviewers. Any product that may be evaluated in this article, or claim that may be made by its manufacturer, is not guaranteed or endorsed by the publisher.

## References

[1] Lundstr MU Wahman K Seiger Å Gray DB Isaksson G. Participation in activities and secondary health complications among persons aging with traumatic spinal cord injury. Spinal Cord. (2017) 55:367–72. 10.1038/sc.2016.153 27845357

[2] Sekhon LH Fehlings MG. Epidemiology, demographics, and pathophysiology of acute spinal cord injury. Spine. (2001) 26:S2–12. 10.1097/00007632-200112151-00002 11805601

[3] Gorgey AS Dudley GA. Skeletal muscle atrophy and increased intramuscular fat after incomplete spinal cord injury. Spinal Cord. (2007) 45:304–9. 10.1038/sj.sc.3101968 16940987

[4] Kocina P. Body composition of spinal cord injured adults. Sports Med. (1997) 23:48–60. 10.2165/00007256-199723010-00005 9017859

[5] Williams PE Goldspink G. Changes in sarcomere length and physiological properties in immobilized muscle. J Anat. (1978) 127:459–68. 744744 PMC1235732

[6] Ciciliot S Rossi AC Dyar KA Blaauw B Schiaffino S. Muscle type and fiber type specificity in muscle wasting. Int J Biochem Cell Biol. (2013) 45:2191–9. 10.1016/j.biocel.2013.05.016 23702032

[7] Pelletier CA Hicks AL. Muscle fatigue characteristics in paralyzed muscle after spinal cord injury. Spinal Cord. (2011) 49:125–30. 10.1038/sc.2010.62 20531355

[8] Scherbakov N Sandek A Doehner W. Stroke-related sarcopenia: specific characteristics. J Am Med Dir Assoc. (2015) 16:272–6. 10.1016/j.jamda.2014.12.007 25676847

[9] Masi AT Hannon JC. Human resting muscle tone (HRMT): narrative introduction and modern concepts. J Bodyw Mov Ther. (2008) 12:320–32. 10.1016/j.jbmt.2008.05.007 19083691

[10] Von Werder SC Disselhorst-Klug C. The role of biceps brachii and brachioradialis for the control of elbow flexion and extension movements. J Electromyogr Kinesiol. (2016) 28:67–75. 10.1016/j.jelekin.2016.03.004 27061680

[11] Krause P Szecsi J Straube A. Changes in spastic muscle tone increase in patients with spinal cord injury using functional electrical stimulation and passive leg movements. Clin Rehabil. (2008) 22:627–34. 10.1177/0269215507084648 18586814

[12] Van Deun B Hobbelen JS Cagnie B Van Eetvelde B Van Den Noortgate N Cambier D. Reproducible measurements of muscle characteristics using the MyotonPRO device: comparison between individuals with and without paratonia. J Geriatr Phys Therapy. (2016) 41:194–203. 10.1519/JPT.0000000000000119 28005829

[13] Gavronski G Veraksits A Vasar E Maaroos J. Evaluation of viscoelastic parameters of the skeletal muscles in junior triathletes. Physiol Meas. (2007) 28:625–37. 10.1088/0967-3334/28/6/002 17664617

[14] Gapeyeva H Vain A. Methodological Guide: Principles of Applying Myoton in physIcal Medicine and Rehabilitation. Tartu: Muomeetria Ltd. (2008).

[15] Marusiak J Kisiel-Sajewicz K Jask Lska A Jask Lski A. Higher muscle passive stiffness in Parkinson's disease patients than in controls measured by myotonometry. Arch Phys Med Rehabil. (2010) 91:800–2. 10.1016/j.apmr.2010.01.012 20434620

[16] Hlich-Zwahlen AK Casartelli NC Item-Glatthorn JF Maffiuletti NA. Validity of resting myotonometric assessment of lower extremity muscles in chronic stroke patients with limited hypertonia: a preliminary study. J Electromyogr Kinesiol. (2014) 24:762–9. 10.1016/j.jelekin.2014.06.007 25023163

[17] Lo WLA Zhao JL Chen L Lei D Huang DF Tong KF. Between-days intra-rater reliability with a hand held myotonometer to quantify muscle tone in the acute stroke population. Sci Rep. (2017) 7:14173. 10.1038/s41598-017-14107-3 29074974 PMC5658427

[18] Lo WLA Zhao JL Li L Mao YR Huang DF. Relative and absolute interrater reliabilities of a hand-held myotonometer to quantify mechanical muscle properties in patients with acute stroke in an inpatient ward. Biomed Res Int. (2017) 2017:4294028. 10.1155/2017/4294028 29164148 PMC5661069

[19] Lo WLA Lei D Leng Y Huang H Wang B Yu Q. Impact of nonsurgical spinal decompression on paraspinal muscle morphology and mechanical properties in young adults with low back pain. J Int Med Res. (2020) 48:300060520919232. 10.1177/0300060520919232 32723102 PMC7391436

[20] Lo WLA Yu Q Mao Y Li W Hu C Li L. Lumbar muscles biomechanical characteristics in young people with chronic spinal pain. BMC Musculoskelet Disord. (2019) 20:559. 10.1186/s12891-019-2935-z 31759390 PMC6875033

[21] Chang TT Feng YN Zhu Y Liu CL Wang XQ Zhang ZJ. Objective assessment of regional stiffness in achilles tendon in different ankle joint positions using the MyotonPRO. Med Sci Monit. (2020) 26:e926407. 10.12659/MSM.926407 33071278 PMC7583434

[22] Rutkove SB. Electrical impedance myography: Background, current state, future directions. Muscle Nerve. (2009) 40:936–46. 10.1002/mus.21362 19768754 PMC2824130

[23] Rutkove SB Aaron R Shiffman CA. Localized bioimpedance analysis in the evaluation of neuromuscular disease. Muscle Nerve. (2002) 25:390–7. 10.1002/mus.10048 11870716

[24] Li J Geisbush TR Rosen GD Lachey J Mulivor A Rutkove SB. Electrical impedance myography for the *in vivo* and *ex vivo* assessment of muscular dystrophy (mdx) mouse muscle. Muscle Nerve. (2014) 49:829–35. 10.1002/mus.24086 24752469 PMC5582805

[25] Li L Shin H Stampas A Li X Zhou P. Electrical impedance myography changes after incomplete cervical spinal cord injury: An examination of hand muscles. Clin Neurophysiol. (2017) 128:2242–7. 10.1016/j.clinph.2017.08.027 29024874

[26] Li L Stampas A Shin H Li X Zhou P. Alterations in localized electrical impedance myography of biceps brachii muscles paralyzed by spinal cord injury. Front Neurol. (2017) 8:253. 10.3389/fneur.2017.00253 28676786 PMC5476999

[27] Rutkove SB Partida RA Esper GJ Aaron R Shiffman CA. Electrode position and size in electrical impedance myography. Clin Neurophysiol. (2005) 116:290–9. 10.1016/j.clinph.2004.09.002 15661107

[28] Buendia R Gil-Pita R Seoane F. Cole parameter estimation from the modulus of the electrical bioimpeadance for assessment of body composition. A full spectroscopy approach. J Electr Bioimped. (2011) 2:72–8. 10.5617/jeb.197

[29] Aird L Samuel D Stokes M. Quadriceps muscle tone, elasticity and stiffness in older males: Reliability and symmetry using the MyotonPRO. Arch Gerontol Geriatr. (2012) 55:e31–9. 10.1016/j.archger.2012.03.005 22503549

[30] Schneider S Peipsi A Stokes M Knicker A Abeln V. Feasibility of monitoring muscle health in microgravity environments using Myoton technology. Med Biol Eng Comput. (2015) 53:57–66. 10.1007/s11517-014-1211-5 25331739

[31] Perell K Scremin A Scremin O Kunkel C. Quantifying muscle tone in spinal cord injury patients using isokinetic dynamometric techniques. Paraplegia. (1996) 34:46–53. 8848323 10.1038/sc.1996.8

[32] Castro MJ Apple DF Staron RS Campos G Dudley GA. Influence of complete spinal cord injury on skeletal muscle within 6 mo of injury. J Appl Physiol. (1999) 86:350–8. 10.1152/jappl.1999.86.1.350 9887150

[33] Shiffman CA Aaron R Amoss V Therrien J Coomler K. Resistivity and phase in localized BIA. Phys Med Biol. (1999) 44:2409–29. 10.1088/0031-9155/44/10/304 10533919

[34] Shah PK Gregory CM Stevens JE Pathare NC Jayaraman A Behrman AL. Non-invasive assessment of lower extremity muscle composition after incomplete spinal cord injury. Spinal Cord. (2008) 46:565–70. 10.1038/sc.2008.10 18347608

[35] Leng Y Lo W Hu CP Bian RH Xu ZQ Shan XY. The effects of extracorporeal shock wave therapy on spastic muscle of the wrist joint in stroke survivors: Evidence from neuromechanical analysis. Front Neurosci. (2021) 14:580762. 33551718 10.3389/fnins.2020.580762 PMC7859269

[36] Li J Staats WL Spieker A Sung M Rutkove SB. A technique for performing electrical impedance myography in the mouse hind limb: data in normal and ALS SOD1 G93A animals. PLoS ONE. (2012) 7:e45004. 10.1371/journal.pone.0045004 23028733 PMC3460964

[37] Wu JS Li J Greenman RL Bennett D Geisbush T Rutkove SB. Assessment of aged mdx mice by electrical impedance myography and magnetic resonance imaging. Muscle Nerve. (2015) 52:598–604. 10.1002/mus.24573 25597760

[38] Dodge Y. Type II Error. The Concise Encyclopedia of Statistics. New York, NY: Springer (2008).

[39] Kortman HG Wilder SC Geisbush TR Narayanaswami P Rutkove SB. Age- and gender-associated differences in electrical impedance values of skeletal muscle. Physiol Meas. (2013) 34:1611–22. 10.1088/0967-3334/34/12/1611 24165434 PMC3895401

[40] Viir R Virkus A Laiho K Rajaleid K Selart A Mikkelsson M. Trapezius muscle tone and viscoelastic properties in sitting and supine positions. SJWEH Suppl. (2007) 2007:76–80.

